# Cyclooxygenase-2 expression is positively associated with lymph node metastasis in nasopharyngeal carcinoma

**DOI:** 10.1371/journal.pone.0173641

**Published:** 2017-03-16

**Authors:** Gui Yang, Qiaoling Deng, Wei Fan, Zheng Zhang, Peipei Xu, Shihui Tang, Ping Wang, Jun’e Wang, Mingxia Yu

**Affiliations:** 1 Department of Clinical Laboratory, Zhongnan Hospital of Wuhan University, Wuhan, Hubei, China; 2 Department of Clinical Laboratory & Center for gene diagnosis, Zhongnan Hospital of Wuhan University, Wuhan, Hubei1, China; 3 Department of Pathology, Zhongnan Hospital of Wuhan University, Wuhan, Hubei, China; University of South Alabama Mitchell Cancer Institute, UNITED STATES

## Abstract

**Background:**

Accumulating evidence has demonstrated that cyclooxygenase-2 (COX-2) is involved in head and neck cancers, especially in nasopharyngeal carcinoma (NPC). However, the association between COX-2 expression and lymph node metastasis in NPC remains uncertain. This systematic review and meta-analysis meta-analysis investigated the relationship between COX-2 expression and lymph node metastasis and other signs of disease progression in NPC.

**Methods:**

Previously published studies assessing COX-2 expression and lymph node metastasis in NPC were identified in four English databases and three Chinese ones (Pubmed, Embase, Cochrane Database of Systematic Reviews, Web of Science, China National Knowledge Infrastructure, Wanfang, Vip Journal Integration Platform) up to November 2016. Quality of all eligible studies was assessed using the Newcastle-Ottawa Quality Assessment Scale (NOS). Pooled odds ratios (OR) and their 95% confidence intervals (95%CI) were calculated with fixed-effects or random-effects model to evaluate the effects of COX-2 expression on lymph node metastasis.

**Results:**

A total of 27 studies with 1797 NPC patients met the inclusion criteria. The expression of COX-2 was significantly higher in patients with nasopharyngeal carcinoma than those without the carcinoma, with a combined OR of 21.17 (95%CI = 15.02–29.85, I^2^ = 35.1%, P_heterogeneity_ = 0.070). A statistically significant association between COX-2 expression and lymph node metastasis in NPC patients, with an OR of 4.44 (95%CI = 3.46–5.70, I^2^ = 38.3%, P_heterogeneity_ = 0.024), and with other indicators of disease progression. Subgroup analyses based on COX-2 assay and staging criteria of TNM showed no significant heterogeneity.

**Conclusions:**

The results suggest that expression of COX-2 is associated with lymph node metastasis and disease progression in NPC, indicating a potential role in evaluation of prognosis and in treatment decisions. COX-2 inhibitors have potential in the treatment of NPC that should be further investigated.

## Introduction

Nasopharyngeal carcinoma (NPC) is an epithelial malignancy arising from the epithelium of the nasopharynx[1]. It is most common in Southeast Asia, especially in Southern China, and parts of North Africa, and is also seen among indigenous Eskimos living in Greenland and Alaska[2]. Epidemiological studies have suggested that environmental and lifestyle factors, including alcohol, diet and tobacco smoking, play an important role in the etiology of NPC, along with the Epstein–Barr virus (EBV)[3, 4].

The American Joint Committee on Cancer (AJCC) TNM classification of malignant tumors, based on anatomical information, is currently the most commonly used staging system and is used to determine treatment regimens for NPC patients[5]. NPC can invade tissues adjacent to the nasopharynx and even metastasize via blood or lymphatic system to bone and organs such as the liver and lungs[6]. A high proportion (70%-80%) of NPC patients present with cervical lymph node metastases when they are first diagnosed. Early lymph node metastasis and the high incidence rate of distant metastases are responsible for 15% to 42% of treatment failures and represent a significant problem[7].

Increasingly research has recognized that inflammation and the inflammatory microenvironment play an important role in cancer development. Cyclooxygenases, as inflammatory regulators, are responsible for the conversion of arachidonic acid to prostaglandin H_2_ (PGH_2_)_._ While one COX isoform, cyclooxygenase-1 (COX-1), is constitutively expressed in most normal tissues and involved in physiological processes under most circumstances[8], a second, cyclooxygenase-2 (COX-2), is an inducible type activated by inflammation or carcinogenic factors. COX-2 can be rapidly induced by mitogenic and inflammatory stimuli, but is usually absent in most normal tissues[9].

Research has shown that COX-2 plays an important role in the carcinogenesis of head and neck cancers (HNC), and in the progression of cancers through modulating cell proliferation and apoptosis in ways that favor tumor growth and metastasis, thus affecting the efficacy of therapies. Therefore, the expression of COX-2 has been proposed as a potential prognostic indicator for prediction of survival in HNC patients, while COX-2 inhibitors may have potential functions as therapeutic agents [10, 11].

However, whereas some studies have concluded that the levels of COX-2 increase when lymph node metastasis occurs in NPC, others have failed to find significant correlations. With insufficient evidence on the association between COX-2 expression and NPC advancement, COX-2’s diagnostic and prognostic values and the role of COX-2 inhibitors in clinical practice remain uncertain.

The purpose of this study was to further investigate the relationship between COX-2 expression and NPC advancement, in particular lymph node metastasis, by systematic review and meta-analysis of existing evidence.

## Methods

This systematic review and meta-analysis was conducted according to the guidelines of the Preferred Reporting Items for Systematic reviews and Meta-Analyses (PRISMA) statement [12].

### Inclusion and exclusion criteria

Studies fulfilling the following criteria were eligible for inclusion:

1. study subjects were human; no restrictions were imposed on the number of samples included in studies;
2. methods used to examine COX-2 expression were immunohistochemistry (IHC) or reverse transcription-polymerase chain reaction (RT-PCR) or real-time polymerase chain reaction (real time PCR);
3. histologically proven NPC patients were distinguishable in two groups: lymph node metastasis group (LNM) and non-lymph node metastasis group (NLNM);
4. the relationship between COX-2 expression and lymph node metastasis in NPC patient were evaluated and Odds Ratio (OR) and its 95% confidence intervals (CI) for the COX-2-positive or COX-2 high expression rate in the two groups were respectively reported; or else, sufficient data were reported for their calculation;
5. studies published in English or Chinese language with full text. No restrictions were imposed on region.

If more than one published article by the same authors or group was identified reporting the same data set, the latest article was included. Studies were excluded that considered potentially overlapping study populations, treated cell lines as research objects were performed in vitro, or lacked necessary information for the calculation of ORs. Conference abstracts were also excluded as these seldom provide sufficient information to assess methodology.

### Publication search

Literature searches were conducted in Embase, PubMed, Cochrane Database of Systematic Reviews, Web of Science, China National Knowledge Infrastructure (CNKI), Wanfangdata and Vip Journal Integration Platform (VIP) for potentially relevant articles published up to November 2016. Search strategy included the following terms: ((Cyclooxygenase-2) OR (COX-2) OR (PTGS2)) AND ((Nasopharyngeal cancer) OR (Nasopharyngeal carcinoma) OR (Nasopharyngeal neoplasm) OR (Nasopharyngeal tumor) OR (NPC)). Additional pertinent studies were sought through hand searching the bibliographies of identified studies and related meta-analyses. Studies identified by the searches were collated in EndNote X7. After excluding duplicates, titles and abstracts were screened independently by two investigators to remove citations that were clearly ineligible. The full texts of potentially eligible studies were obtained and reviewed in detail independently by the two reviewers. At both stages any disagreements about selection were discussed by the two reviewers to reach a consensus according to the inclusion and exclusion criteria.

### Data extraction

From each eligible article, two investigators independently abstracted information about the first author's name, year of publication, study country, methods for COX-2 detection, COX-2 positivity, TNM malignant tumor classification, clinical stage, metastasis status LNM (N0) and NLNM (N1, N2, N3), metastasis stage, tumor size stage, differentiation types and sex. For studies that presented both univariate and multivariate analyses, data were extracted from the latter type because such analyses take into account potential influences of predictive or confounding factors.

### Quality of eligible studies

The quality of the included studies was evaluated using the Newcastle-Ottawa Scale (NOS) [13] by two reviewers independently. The stars rating system with a highest score of 9 considers three domains: selection of the study population; comparability and exposure assessment.

### Statistical methods

For all analyses, non-lymph node metastases N1, N2 and N3 were combined; malignant tumor classification T1 and T2 were combined, as were T3 and T4; clinical stage I and stage II were combined, as were stage III and stage IV; high differentiation and moderate differentiation were combined, as were low differentiation and dedifferentiation. As the AJCC and UICC NPC staging systems are similar, they were combined for principal analyses. OR was obtained as A vs B. Pooled OR and 95%CI were calculated based on primary data from the selected studies. An OR > 1 implied a higher susceptibility of lymph node metastasis in COX-2-positive NPC patients when 95%CIs did not include 1 (p < 0.05). Statistical heterogeneity among studies was evaluated with the Cochran Chi square-based Q-test and I square test. All tests were two-tailed, and heterogeneity was considered statistically significant when the Cochran Q test p value was less than 0.10 and I^2^ value was greater than 50%. We combined OR using fixed-effects model (the Mantel-Haenszel method) unless significant heterogeneity was indicated, in which case random-effects model (the DerSimonian-Laired method) was used. Sensitivity analysis was conducted by sequentially omitting selected study to evaluate their effects on pooled OR and 95% CI. Subgroup analyses were conducted to consider variations in findings according to assays used, NPC staging systems used and sex, tumor size stage, metastasis stage, clinical stage and histological differentiation stage. Begg's funnel plot was created to estimate potential publication bias and Egger's test was used to check the symmetry of funnel plots. We considered publication bias to be negligible when p < 0.05 from Egger’s test. All analyses were performed using Stata14.0 Statistical Software.

## Results

### Selection of studies

**Fig 1:**
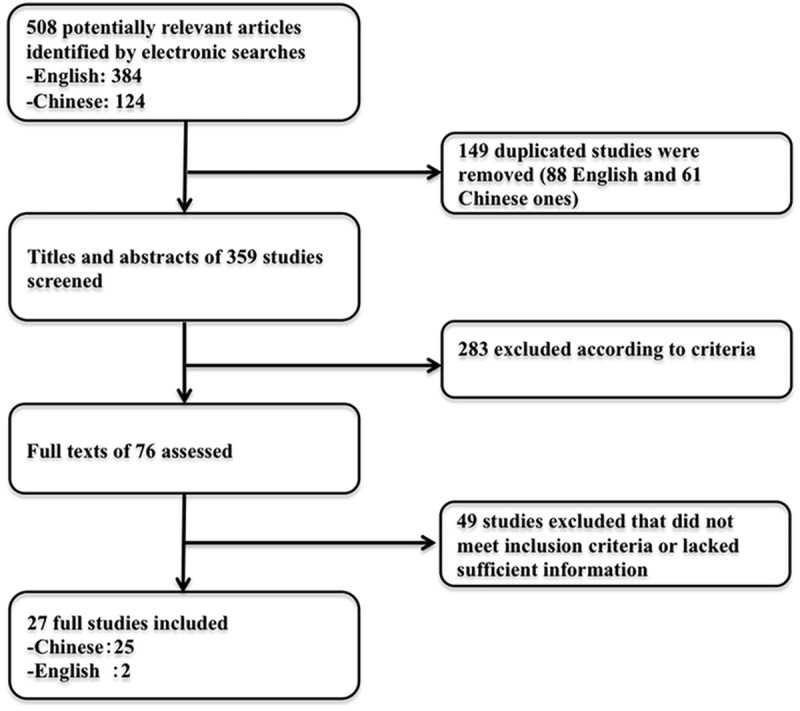
Flow diagram of articles selection.

A total of 508 potentially relevant studies were identified by our search strategy. After 149 duplicates were removed, the titles and abstracts of the remaining 359 were screened and 283 studies rejected. After full text assessment, 49 papers that did not meet the eligibility criteria or lacked essential information were rejected, so that 27 studies published after 2004 with 1797 NPC patients were included in our analysis. A flow diagram of article selection is given in Fig 1.

### Characteristics of included studies

The main characteristics of the 27 included studies are summarized in Table 1. Two studies were written in English[14, 15], while the rest were Chinese articles[16–40]. Among the 1797 cases in the included studies, there were 1265 patients with lymph node metastasis and 532 NPC patients without metastasis, and 1346 cases with COX-2 expression and 451 cases without. The cumulative lymph node metastasis rate and COX-2-positive rate of NPC were 70.40% (1265/1797) and 74.90% (1346/1797), respectively. The cumulative COX-2 expression rates were 82.69% (1046/1265) in the LNM group and 56.39% (300/532) in the NLNM group (Table 2). Immunohistochemistry (IHC) was the main method for the evaluation of COX-2 expression in NPC specimens, with 17 studies using IHC-SP and another 5 studies using IHC-SABC. The remaining studies did not reporting detailed information on which IHC assay was used. One study assessed COX-2 mRNA expression with RT–PCR and one with both IHC and real-time PCR. Seven studies used the AJCC/UICC staging system, while seven used the 1992 Chinese staging system. Methodological appraisals of the included studies using the Newcastle Ottawa scale are presented in Table 3.

**Table 1:** Main characteristics of included studies.

First author-Year	Country	COX-2 assay	Classification	Patients	Control	Age	NLNM	LNM
**Xu Xinhua-2006**	China	IHC-SP	China 1992	86(63)	10(0)	Median 46	10(4)	76(59)
**Jiang Daihua-2004**	China	IHC-SABC	-	62(48)	10(0)	Mean 48.98	18(17)	44(31)
**Gu Shanzhi-2006**	China	IHC-SP	China 1992	78(40)	-	Median 46	24(5)	54(35)
**Zhou Leyuan-2006**	China	IHC	China 1992	43(34)	-	Mean 54.9	7(5)	36(29)
**Dou Yanling-2006**	China	IHC-SABC	-	39(35)	-	Mean 51.2	20(16)	19(19)
**Luo Weireng-2006**	China	IHC-SP	-	95(65)	26(7)	-	65(40)	30(25)
**Fu Yafeng-2007**	China	IHC	China 1992	53(46)	23(10)	-	21(17)	32(29)
**Zhou Fang-2007**	China	IHC-SP	-	45(32)	20(2)	Mean 60.3	17(9)	28(23)
**Zhu Hongyuan-2008**	China	IHC-SP	AJCC	56(43)	20(3)	-	12(6)	44(37)
**Miao Beiping-2007**	China	IHC-SP	China 1992	60(54)	30(2)	-	22(17)	38(37)
**Gong Yongqian-2007**	China	IHC-SP	UICC	97(83)	20(4)	-	28(18)	69(65)
**Cui Dewei-2008**	China	IHC-SP	-	86(65)	30(1)	-	25(10)	61(55)
**Yuan Hong-2008**	China	IHC-SP	UICC	45(31)		Median 44	17(7)	28(24)
**Xie Zhenyu-2008**	China	IHC-SP	China 1992	52(33)	20(4)	Median 45.8	19(8)	33(25)
**Zhang Tingyou-2009**	China	IHC-SABC	China 1992	20(14)		Mean 44.8	5(4)	15(10)
**Xiong Danning—2009**	China	IHC-SP	-	86(69)	-	Mean 43.3	32(24)	54(45)
**Liu Yangyun-2010**	China	IHC-SP	AJCC	50(32)	15(0)	-	15(4)	35(28)
**Jing Qiancheng-2010**	China	IHC-SP	UICC	33(27)	12(3)	Mean 49.8	13(8)	20(19)
**Wu Jingbo-2011**	China	IHC		45(35)	20(2)	Mean 58.6	14(7)	31(28)
**Wang Zhiyong-2011**	China	IHC-SP	-	66(52)	20(2)	-	26(14)	40(38)
**Xi Shaoyan-2012**	China	IHC-SP	AJCC/UICC	86(61)	-	Mean 45	7(5)	79(56)
**Li Jianping-2012**	China	RT-PCR	-	32(22)	11(3)	-	17(9)	15(13)
**Bai Weiqi-2012**	China	IHC	UICC	58(46)	38(3)	Mean 47	18(8)	40(38)
**Zhang Daqun-2013**	China	IHC	-	76(56)	32(2)	Mean 57.9	21(10)	55(46)
**Zhu Honghai-2014**	China	IHC	-	104(82)	-	Mean 48.7	20(11)	84(71)
**Dingbo Shi-2014**	China	IHC	-	200(138)	-	-	37(17)	163(121)
**Ali Fendri-2008**	Tunisia	Real Time-PCR	-	44(40)	10(2)	Media 40	2(0)	42(40)
**Overall**	-	-	-	1797(1346)	-	-	532(300)	1265(1046)

Abbreviations: NLMN: non-lymph node metastasis; LMN: lymph node metastasis; IHC: immunohistochemistry; SP: streptavidin-peroxidase; SABC: streptavidin avidin-biotin-pcroxidase complex method; RT-PCR: real time polymerase chain reaction; AJCC: The American Joint Committee on Cancer; UICC: Union for International Cancer Control.

**Table 2:** Other clinical characteristics of included studies.

First author -Year	Gender		Tumor size stage		Distance metastasis		TNM stage		Differential	
	Male(+)	Female(+)	T1/T2(+)	T3/T4(+)	M0(+)	M1(+)	I/II(+)	III/IV(+)	High(+)	Low(+)
Xu Xinhua-2006	65	21	-	-	-	-	-	-	11(5)	75(58)
Jiang Daihua-2004	49(38)	13(10)	-	-	-	-	-	-	-	-
Gu Shanzhi-2006	61	17	38(15)	40(29)	73(39)	5(5)	-	-	-	-
Zhou Leyuan-2006	32(27)	11(7)	-	-	-	-	11(6)	32(29)	-	-
Dou Yanling-2006	28	11	-	-	20	19	11(4)	28(25)	-	-
Luo Weireng-2006	-	-	-	-	-	-	-	-	-	-
Fu Yafeng-2007	36(33)	17(13)	52(45)	1(1)	-	-	39(32)	14(14)	-	-
Zhou Fang-2007	31	14	29(19)	16(13)	-	-	-	-	-	-
Zhu Hongyuan-2008	-	-	-	-	-	-	15(8)	41(35)	-	-
Miao Beiping-2007	-	-	-	-	-	-	25(20)	35(34)	29(24)	31(30)
Gong Yongqian-2007	71(61)	26(22)	-	-	-	-	44(33)	53(50)	-	-
Cui Dewei-2008	-	-	-	-	-	-	-	-	-	-
Yuan Hong-2008	35(24)	10(7)	-	-	-	-	18(9)	27(22)	-	-
Xie Zhenyu-2008	41(26)	11(7)	-	-	-	-	17(7)	35(26)	-	-
Zhang Tingyou-2009	15	5	10(7)	10(7)	18(6)	2(2)	6(4)	14(12)	-	-
Xiong Danning—2009	64(53)	22(16)	-	-	-	-	38(33)	48(46)	-	-
Liu Yangyun-2010	25(18)	25(15)	14(3)	36(30)	-	-	-	-	-	-
Jing Qiancheng-2010	25(20)	8(7)	15(10)	18(17)	-	-	-	-	2(1)	31(26)
Wu Jingbo-2011	32(25)	13(10)	-	-	-	-	-	-	-	-
Wang Zhiyong-2011	51(40)	15(12)		-	-	-	-	-	19(12)	47(40)
Xi Shaoyan-2012	61(40)	21(21)	45(30)	41(31)	-	-	40(25)	46(36)	-	-
Li Jianping-2012	-	-	-	-	-	-	-	-	-	-
Bai Weiqi-2012	40(31)	18(15)	-	-	-	-	19(10)	39(36)	-	-
Zhang Daqun-2013	53	23	31(24)	45(32)	-	-	-	-	-	-
Zhu Honghai-2014	85(67)	19(15)	-	-	-	-	-	-	-	-
Dingbo Shi-2014	145(101)	55(37)	76(41)	124(97)	153(57)	47(42)	78(36)	122(102)	-	-
Ali Fendri-2008	30	15	-	-	-	11	-	-	-	-

**Table 3:** Assessment of study quality according to Newcastle-Ottawa Scale (NOS).

Studies	Items									NOS score
	1	2	3	4	5a	5b	6	7	8	
Xu Xinhua-2006	*	*	*	-	-	-	*	*	-	5
Jiang Daihua-2004	*	*	*	-	-	-	*	*	-	5
Gu Shanzhi-2006	*	*	*	*	-	-	*	*	-	6
Zhou Leyuan-2006	*	*	*	-	-	-	*	*	-	5
Dou Yanling-2006	*	*	*	-	-	-	*	*	-	5
Luo Weireng-2006	*	*	*	-	-	-	*	*	-	5
Fu Yafeng-2007	*	*	*	-	-	-	*	*	-	5
Zhou Fang-2007	*	*	*	-	-	-	*	*	-	5
Zhu Hongyuan-2008	*	*	*	*	-	-	*	*	-	6
Miao Beiping-2007	*	*	*	-	-	-	*	*	-	5
Gong Yongqian-2007	*	*	*	-	-	-	*	*	-	5
Cui Dewei-2008	*	*	*	-	-	-	*	*	-	5
Yuan Hong-2008	*	*	*	-	-	-	*	*	-	5
Xie Zhenyu-2008	*	*	*	-	-	-	*	*	-	5
Zhang Tingyou-2009	*	*	*	-	-	-	*	*	-	5
Xiong Danning—2009	*	*	*	-	-	-	*	*	-	5
Liu Yangyun-2010	*	*	*	-	-	-	*	*	-	5
Jing Qiancheng-2010	*	*	*	-	-	-	*	*	-	5
Wu Jingbo-2011	*	*	*	-	-	-	*	*	-	5
Wang Zhiyong-2011	*	*	*	-	-	-	*	*	-	5
Xi Shaoyan-2012	*	*	*	-	-	-	*	*	-	5
Li Jianping-2012	*	*	*	-	-	-	*	*	-	5
Bai Weiqi-2012	*	*	*	-	-	-	*	*	-	5
Zhang Daqun-2013	*	*	*	-	-	-	*	*	-	5
Zhu Honghai-2014	*	*	*	*	-	-	*	*	-	6
Dingbo Shi-2014	*	*	*	-	-	-	*	*	-	5
Ali Fendri-2008	*	-	*	*	-	-	*	*	-	5

Annotations: 1 adequacy of the case definition; 2 representativeness of the cases; 3 selection of controls; 4 definition of controls. 5a multiple ratings for this item for different categories of exposure; 5b other controlled factors; 6 ascertainment of exposure; 7 same method of ascertainment for cases and controls; 8 non-response rate.

### Quantitative synthesis

#### COX-2 expression in NPC and non-NPC tissues

**Fig 2:**
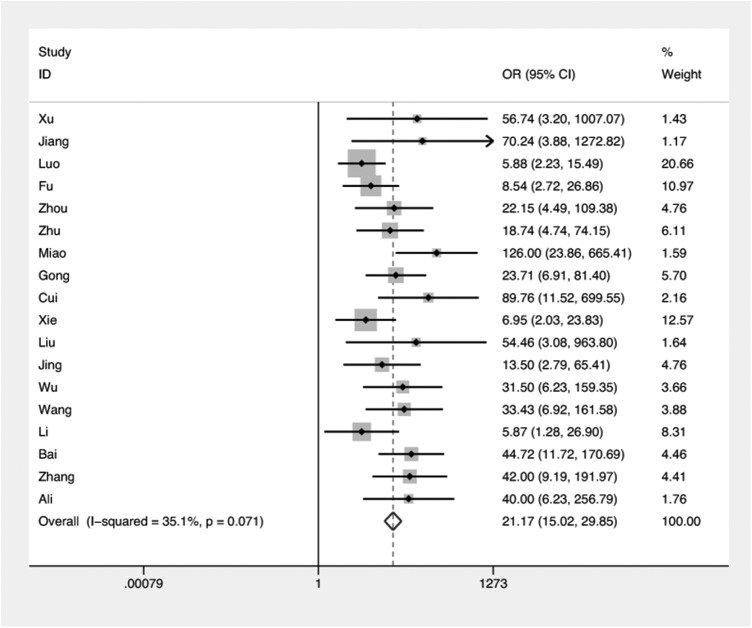
Forest plot: odds ratio (OR) for COX-2 expression in NPC and normal tissue.

Data from 18 studies were included in meta-analysis comparing COX-2 expression in NPC and non-NPC tissues (Fig 2). No significant between-study heterogeneity was detected across these studies and therefore a fixed-effects model was used to calculate a combined OR. Overall, there was a significant association between COX-2 expression and NPC (OR = 21.17, 95%CI = 15.02–29.85, P_heterogeneity_ = 0.07).

#### COX-2 expression and lymph node metastasis in NPC

**Fig 3:**
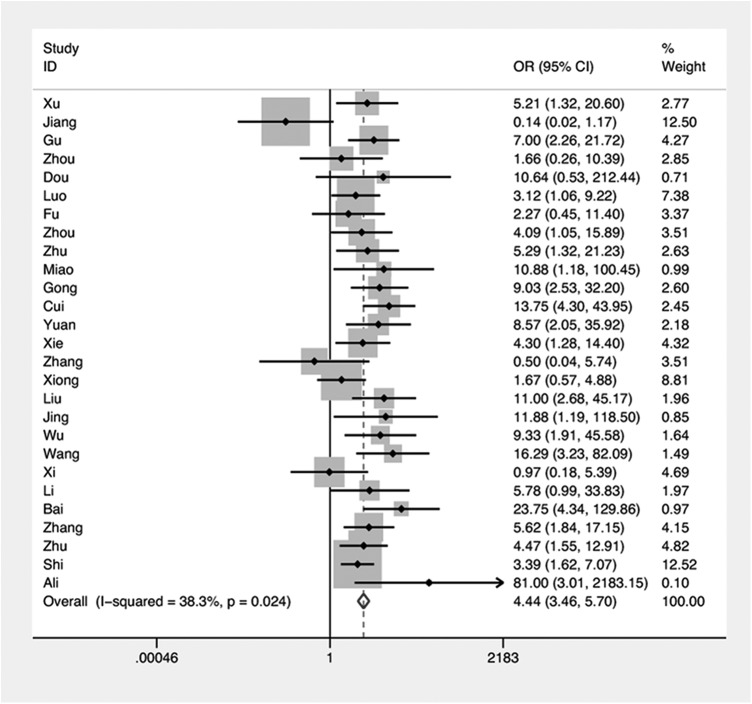
Forest plot:odds ratio (OR) for COX-2 expression and lymph node metastasis in NPC.

All 27 studies were included in a meta-analysis that determined a statistically significant association between COX-2 expression and lymph node metastasis in NPC (OR = 4.44, 95%CI = 3.46–5.70) (Fig 3). Considered individually, of 24 studies with ORs >1, 19 reported 95%CIs not including 1, indicating a positive correlation between lymph node metastasis of NPC and COX-2 expression; only 3 studies reached ORs <1. As the heterogeneity test showed no significant heterogeneity among studies (I^2^ = 38.3%, P_hetrogeneity_ = 0.024), a fixed-effects model was used. Subgroup analysis by assay and staging criteria also provided evidence for an association between COX-2 and lymph node metastasis in NPC patients (OR_IHC-SP_ = 6.29, 95%CI = 4.55–8.67; OR_IHC-SABC_ = 2.42, 95%CI = 1.45–4.02)(Table 4).

**Table 4:** Main results from subgroup analyses.

Subtypes	No.	OR	95% CI	Z (p)	P_heterogeneity_
**Assay**					
**IHC-SP**	17	6.29	4.55,8.67	11.19(0.000)	0.561
**IHC-SABC**	5	2.42	1.45,4.02	3.40(0.001)	0.006
**IHC-unclear**	3	1.80	0.81,4.01	1.44(0.150)	0.947
**RT-PCR**	1	5.78	0.99,33.83	1.95(0.052)	-
**RT-PCR&** **IHC-SABC**	1	81.00	3.01,2183.15	2.61(0.009)	-
**Staging criteria**					
**China 1992**	7	3.98	2.30,6.86	4.96(0.000)	0.421
**AJCC/UICC**	7	7.26	4.22,12.48	7.17(0.000)	0.24
**Unknown**	13	3.88	2.80,5,39	8.12(0.000)	0.014
**Country**					
**China**	26	4.37	3.40,5.61	11.55(0.000)	0.035
**Tunisia**	1	81.00	3.01,2183.15	2.61(0.000)	-
**Overall**	27	4.44	3.46,5.70	11.74(0.000)	0.024

Abbreviations: IHC: immunohistochemistry; SP: streptavidin-peroxidase; SABC: streptavidin avidin-biotin-pcroxidase complex method; RT-PCR: real time polymerase chain reaction; AJCC: The American Joint Committee on Cancer; UICC: Union for International Cancer Control.

#### COX-2 expression and other clinicopathological parameters

**Fig 4:**
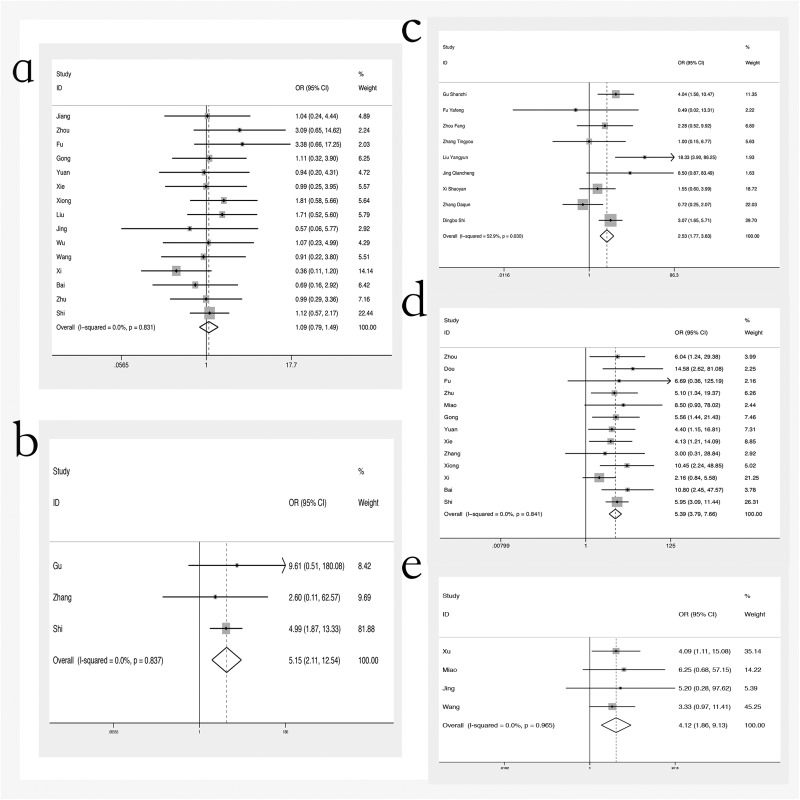
Forest plots: odds ratio (OR) for other clinicopathological parameters. (a) sex (male vs female); (b) metastasis (M1 vs M0); (c) tumor size (T1/T2 vs T3/T4); (d) TNM stage (I/II vs III/IV); (e) differentiation (low vs high)

Additional analyses explored potential associations of COX-2 expression with other potentially relevant factors, such as sex, tumor size stage (T3/T4 vs T1/T2), metastasis stage (M1 vs M0), clinical stage (III/IV vs I/II), and histological differentiation stage (no or low vs medium or high) (Fig 4). No statistically significant association was observed between COX-2 expression and sex (OR 1.09, 95%CI = 0.79–1.49). However, there were statistically significant positive associations of Cox 2 expression with more advanced clinical stages (III/IV) compared to early localized stages (I/II) (OR 5.39, 95%CI = 3.79–7.66), with the presence of more advanced metastases (OR 5.15, 95%CI 2.11–12.54), with larger tumor size (T3/T4) compared to smaller (T1/T2) (OR 2.53, 95%CI 1.77–3.63) and with no/low histological differentiation compared to medium/high (OR 4.12, 95%CI = 1.86–9.13).

#### Sensitivity analysis

**Fig 5:**
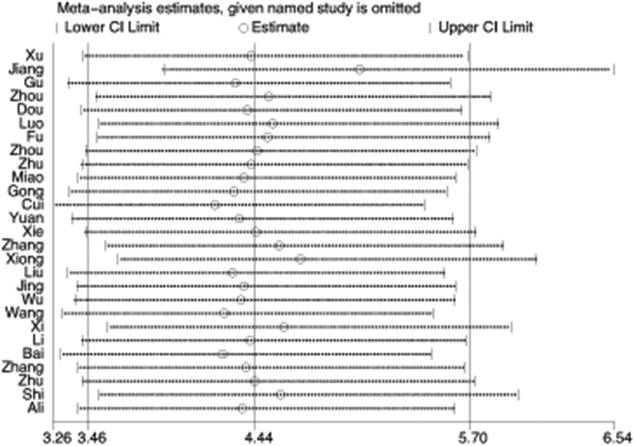
Sensitivity analysis to test the influences of individual studies on overall effects.

The influence of individual studies on pooled results were assessed by conducting sensitivity analysis in which each individual study was omitted in turn and pooled ORs were recomputed and compared with the overall OR. The recalculated ORs did not differ significantly from the overall value, indicating that the overall results were stable (Fig 5).

#### Publication bias

**Fig 6:**
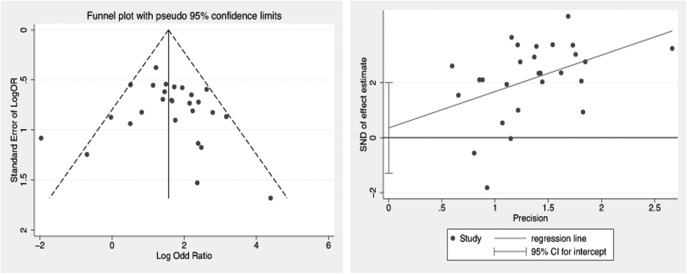
Funnel Plots of publication bias summary for corresponding Meta-analysis (left) and Egger's publication bias plot with pseudo 95%CI (right)

Publication bias was evaluated using funnel plots and Begg’s (P = 0.404) and Egger’s (P = 0.658) tests, and none of them suggested significant bias was present, considering the absence of obvious asymmetry in plot shape and values of p = 0.404 and 0.658, respectively, for the statistical tests (Fig 6).

## Discussion

This systematic review and meta-analysis found that COX-2 expression was positively associated with NPC lymph node metastasis and with other indicators of disease progression.

### Review findings in the context of existing literature

Recent meta-analyses have synthesized evidence on the association between COX-2 expression and various types of cancers[41–44]. While some studies have reported an association between COX-2 levels and lymph node metastasis in NPC, others have found none, and COX-2’s diagnostic and prognostic values in NPC and the role of COX-2 inhibitors in treatment remain uncertain. This new systematic review and meta-analysis demonstrated a clear association of COX-2 expression with NPC-related lymph node metastasis and with other clinicopathological parameters.

The role of COX-2 in oncogenesis has been extensively explored in vitro tests using different tumor cell lines as well as in vivo experiments[45, 46]. Some researchers have suggested that COX-2 is induced by LMP-1 and contributes to the cancer process via the co-expression with VEGF, EGFR or other biomarkers which could enhance several cell survival and proliferation signal pathways[15, 47]. Others suggest that COX-2 expression could promote angiogenesis in NPC [48–50]. Several studies have shown that the anticancer effects of COX-2 inhibitors on NPC cells result from blocking cell cycle and inducing cell apoptosis, which may be partly mediated through the STAT3 pathway or through the inhibition of AKT phosphorylation[51, 52].

Although the precise mechanism by which COX-2 influences cancer development and progression in NPC remains unclear, this study highlights COX-2 expression’s potential as an indicator of cancer progression that can contribute to assessment of prognosis and treatment decisions and the therapeutic potential of COX-2 inhibitors in treatment of NPC.

In pooling and analyzing the data, considerable attention was paid to the methods, characteristics and results of each study individually in order to fully explore possible sources of heterogeneity or other factors that may affect overall results. Detection methods were examined and subgroup analyses conducted to evaluate possible heterogeneity arising from variations in technique and standards for identifying COX-2 positivity. Despite poor standardization of assay methods, no significant heterogeneity was detected resulting from detection methods other than in the 5-study IHC-SABC group. Subgroup analysis found no significant heterogeneity arising from use of either AJCC/UICC or Chinese TNM classification. Between study heterogeneity was not found to be significant in sensitivity analyses in which one study was removed at a time and recalculated ORs compared with the overall ORs.

### Implications for clinical practice

In recent decades, advances have been made in the control of severe local invasion associated with NPC treated with chemoradiotherapy (CRT), sometimes combined with targeted drug therapies, and significantly improvements have been achieved in clinical outcomes for NPC patients[53–58]. However, early lymph node metastasis is still common even in newly diagnosed NPC cases. These metastases reduce the potential of treatments to prevent secondary diseases and disease recurrences, and are the major cause of the low survival rate associated with the disease.

Research has shown that COX-2 plays an important role in the carcinogenesis of head and neck cancers (HNC), and in the progression of cancers through modulating cell proliferation and apoptosis in ways that favor tumor growth and metastasis, thus affecting the efficacy of therapies. Therefore, COX-2 inhibitors may have considerable potential as therapeutic agents.

### Implications for research

Further clinical research is warranted to develop and investigate ways in which COX-2 expression may be used clinically for assessment of disease progression, consideration of prognosis and treatment decisions in NPC. In addition, research is needed to formally explore and assess the therapeutic potential of COX-2 inhibitors in the disease. The data on which this review is based were obtained from studies with retrospective design; well-designed prospective clinical research and randomized controlled trials are needed to verify our findings.

In order to inform clinical developments that may benefit patients, more work is required to better understand the underlying mechanism of the occurrence and development of NPC-related lymph node metastasis, which remains poorly understood. The identification of molecular biomarkers associated with metastasis and recurrence in NPC is important in prognosis prediction, treatment decision-making and the development of therapies for NPC. In recent years, a great deal of research has focused on molecular biomarkers for cancers, including NPC. To date, researchers have investigated the associations of many biomarkers with the occurrence, progression and metastasis of NPC, including EBV-LMP1[59–61], EGFR[51, 62, 63], COX-2[41, 64–68], VEGF[69–73], etc [74–81].

The development of standardized assays for the detection of biomarkers is vital. RT-PCR is the most standardized technique in the detection of COX-2 expression. IHC is commonly used in most studies, as it is affordable and convenient to operate on formation-fixed tissues in retrospective studies. But this manual testing also has its own limitations, including the use of varied primary antibodies with different dilutions, IHC staining protocols with no definitions for the exact pH and compounds of the solutions or heating methods, and diverse and sometimes arbitrary cut-off values, limited samples and other human factors.

### Study limitations

The present study has some limitations that should be noted. Although the quality of included studies as assessed using the NOS were generally adequate individually, few quality scores were high, which lowers the overall reliability of the review’s findings. Some potentially relevant studies, for example, were excluded because they reported insufficient data to extract OR values. In the included studies about the correlation of COX-2 expression with lymph node metastasis, most ORs were determined only by univariate analysis, which might influence the results. Non-Chinese patients accounted for only 2.45% (44/1797) of the total cases, which may be considered to reduce the generalizability of the results. The very high proportion of Chinese studies may reflect higher levels of research activity relating to the clinical area, resulting from the fact that NPC is one of the most common head and neck cancers in Southeast Asia, especially in Southern China. During the conduct of the review, given the overwhelmingly Chinese evidence-base, a second broad search was conducted to assess whether non-Chinese studies had been missed, but none was identified.

## Conclusions

The review has strengthened the evidence for the association between COX-2 expression and lymph node metastasis in nasopharyngeal carcinoma patients, and with other indicators of disease progression. This suggests not only that COX-2 may be involved in the occurrence and development of lymph node metastasis and in disease progression, but also that it may have potential in assessing prognosis and in treatment decisions. In addition, it highlights the potential of COX-2 inhibitors in treatment of NPC. Well-designed prospective studies are needed to develop and investigate methods for using COX-2 expression for more accurate assessment of disease progression, and to assess the therapeutic potential of COX-2 inhibitors as treatment for the disease.

## Supporting information

S1 PRISMA Checklist PRISMA Checklist. (PDF)
